# Supplementary material for: Species divergence in valleys: the phylogeny of Phrynocephalus forsythii complex and description of a new species
Source: PeerJ. 2024 Mar 27;12:e17175. doi: 10.7717/peerj.17175 (PMC10981416; doi:10.7717/peerj.17175)
Supplement: Supplemental Information 1 [file peerj-12-17175-s001.docx]

Supplemental Table 1 Localities, and GenBank accession numbers for all species used in this study.

| **Species Before** | **Species** | **GenBank number** | **Voucher ID** | **Locality** | **E** | **N** | **Source** |
| --- | --- | --- | --- | --- | --- | --- | --- |
| P. forsythii | Phrynocephalus kangsuensis **sp. nov.** | OR878656(COI)  OR884245(16S) | XJA0010100  (CX09243) | China, Xinjiang,Wuqia County, Near kangsu Town (altitude: 2717m) | 74.73 | 39.80 | This study |
| P. forsythii | Phrynocephalus kangsuensis **sp. nov.** | OR878657(COI)  OR884246(16S) | XJA0010101  (CX09244) | China, Xinjiang,Wuqia County, Near kangsuTown (altitude: 2717m) | 74.73 | 39.80 | This study |
| P. forsythii | Phrynocephalus kangsuensis **sp. nov.** | OR878658(COI)  OR884247(16S) | XJA0010102  (CX09245) | China, Xinjiang,Wuqia County, Near kangsuTown (altitude: 2717m) | 74.73 | 39.80 | This study |
| P. forsythii | Phrynocephalus kangsuensis **sp. nov.** | OR878659(COI)  OR884248(16S) | XJA0010103  (CX09246) | China, Xinjiang,Wuqia County, Near kangsu Town (altitude: 2717m) | 74.73 | 39.80 | This study |
| P. forsythii | Phrynocephalus kangsuensis **sp. nov.** | OR878654(COI)  OR884249(16S) | XJA0010104  (CX09247) | China, Xinjiang,Wuqia County, Near kangsuTown (altitude: 2717m) | 74.73 | 39.80 | This study |
| P. forsythii | Phrynocephalus kangsuensis **sp. nov.** | OR878655(COI)  OR884250(16S) | XJA0010105  (CX09248) | China, Xinjiang,Wuqia County, Near kangsu Town (altitude: 2717m) | 74.73 | 39.80 | This study |
| P. forsythii | Phrynocephalus **sp.** | OR875274(COI)  OR875282(16S) | XJA0010106  (CX607) | China, Xinjiang, Hotan County, Near Ulurwati (altitude: 2125m) | 79.45 | 36.78 | This study |
| P. forsythii | Phrynocephalus **sp.** | OR875272(COI)  OR875280(16S) | XJA0010107  (CX600) | China, Xinjiang, Hotan County, Near Ulurwati (altitude: 2125m) | 79.45 | 36.78 | This study |
| P. forsythii | Phrynocephalus **sp.** | OR875273(COI)  OR875281(16S) | XJA0010108  (CX605) | China, Xinjiang, Hotan County, Near Ulurwati (altitude: 2125m) | 79.45 | 36.78 | This study |
| P. forsythii | Phrynocephalus **sp.** | OR875275(COI)  OR875283(16S) | XJA0010109  (XHY01001) | China, Xinjiang, Hotan County, Near Ulurwati (altitude: 2125m) | 79.45 | 36.78 | This study |
| P. forsythii | P. forsythii | OR875264(COI)  OR875284(16S) | XJA0010110  (CX09181) | China, Xinjiang, Aheqi (altitude: 3148m) | 77.34 | 40.82 | This study |
| P. forsythii | P. forsythii | OR875265(COI)  OR875285(16S) | XJA0010111  (CX09182) | China, Xinjiang, Aheqi  (altitude: 3148m) | 77.34 | 40.82 | This study |
| P. forsythii | P. forsythii | OR875266(COI)  OR875286(16S) | XJA0010112  (CX09184) | China, Xinjiang, Aheqi  (altitude: 3148m) | 77.34 | 40.82 | This study |
| P. forsythii | P. forsythii | OR875268(COI)  OR875287(16S) | XJA0010113  (CX09185) | China, Xinjiang, Aheqi  (altitude: 3148m) | 77.34 | 40.82 | This study |
| P. forsythii | P. forsythii | OR875269(COI)  OR875288(16S) | XJA0010114  (CX09186) | China, Xinjiang, Aheqi  (altitude: 3148m) | 77.34 | 40.82 | This study |
| P. forsythii | P. forsythii | OR875267(COI)  OR875289(16S) | XJA0010115  (CX09187) | China, Xinjiang, Aheqi  (altitude: 3148m) | 77.34 | 40.82 | This study |
| P. forsythii | P. forsythii | OR875270(COI)  OR875290(16S) | XJA0010116  (CX09188) | China, Xinjiang, Aheqi  (altitude: 3148m) | 77.34 | 40.82 | This study |
| P. forsythii | P. forsythii | OR875271(COI)  OR875291(16S) | XJA0010117  (CX09189) | China, Xinjiang, Aheqi  (altitude: 3148m) | 77.34 | 40.82 | This study |
| P. forsythii | P. forsythii | KP232959 | - | China, Xinjiang, Aksu  (altitude: 3148m) | 80.9 | 40.42 | Shao *et al.* 2016 |
| P. forsythii | P. forsythii | MK461355 | ZMMU R-14324 | China, Xinjiang, N Takla-Makan, Aksu, 5 km N  Aral | 81.234 | 40.42 | Solovyeva *et al.* 2011 |
| P. forsythii | P. forsythii | KP126516 | WGXG08371 | China, Xinjiang, Qiemo County | - | - | Chen *et al.* 2016 |
| P. forsythii | P. forsythii | AY053784 | CIB0004 | Chian, Xinjiang, Kashi | - | - | Pang *et al.* 2003 |
| P. forsythii | P. forsythii | AY053783 | CIB01097 | Chian, Xinjiang, The Airport of Aksu | - | - | Pang *et al.* 2003 |
| P. forsythii | P. forsythii | AY053782 | CIB01099 | Chian, Xinjiang, The Airport of Aksu | - | - | Pang *et al.* 2003 |
| P. forsythii | P. forsythii | AY053781 | CIB01037 | Chian, Xinjiang, Jamtai of Wensu | - | - | Pang *et al.* 2003 |
| P. forsythii | P. forsythii | AY053780 | CIB01038 | Chian, Xinjiang, Jamtai of Wensu | - | - | Pang *et al.* 2003 |
| P. forsythii | P. forsythii | MK284225 | WGXG08351 | Chian, Xinjiang, Ruoqiang | - | - | Chen *et al.* 2019a |
| *P. nasatus* | P. forsythii | MW456559 | GXG1093 | China, Xinjiang, Baicheng County | - | = | Guo *et al.* 2022 |
| *P. nasatus* | P. forsythii | MK461474 | ZMMU R-14317-4 | China, Xinjiang, Aksu, 75 km NE Aksu, 5.3 km N  from Norerik | 80.833 | 41.743 | Solovyeva *et al.* 2023 |
| *P. nasatus* | P. forsythii | MK461475 | ZMMU R-14317-2 | China, Xinjiang, Aksu, 75 km NE Aksu, 5.3 km N from Norerik | 80.833 | 41.743 | Solovyeva *et al.* 2023 |
| *P. nasatus* | P. forsythii | MK461476 | ZMMU R-14317-3 | China, Xinjiang, Aksu, 75 km NE Aksu, 5.3 km N from Norerik [type locality of nasatus] | 80.833 | 41.743 | Solovyeva *et al.* 2023 |
| *P. vlangalii nanschanica* | *P. nanschanicus* | MF039061 | - | China, Nanshan Mountains, Southern Danghe range, Northern QTP | 96.06 | 38.97 | Jin *et al.* 2018 |
| *P. erythrurus* | *P. vlangalii vlangalii* | KF691718 | ZMMU R-12303 | China, Qinghai, environs of Duokake, KunLun Shan (KunLun mt) | 92.328 | 36.611 | Solovyeva *et al.* 2014 |
| *P. erythrurus* | *P. vlangalii vlangalii* | MK461351 | ZMMU R-12305-1 | China, Qinghai, environs of Golmud, Burhan Budai Shan | 94.19 | 36.43 | Solovyeva *et al.* 2023 |
| *P. erythrurus* | *P. vlangalii vlangalii* | MK461352 | ZMMU R-12304 | China, Xinjiang Uygur Zizhiqu, Aer Jin Shan N. P., Arijin mt | 90.834 | 36.985 | Solovyeva *et al.* 2023 |
| *P. erythrurus* | *P. vlangalii vlangalii* | MK461353 | ZMMU R-12307 | China, 40 km NW from Tangulashan, Kekexili mt, near Kekexili | 92.6 | 34.833 | Solovyeva *et al.* 2023 |
| *P. erythrurus* | *P. vlangalii vlangalii* | MK461354 | ZMMU R-12306 | China, Qinghai, KunLun Shan Kou, environs of Tibetan plato | 93.519 | 35.072 | Solovyeva *et al.* 2023 |
| *P. vlangalii* | *P. vlangalii vlangalii* | MF039058 | - | China, Geermu, Western Qinghai-Xizang (Tibetan) Plateau | - | - | Zhu *et al.* 2016 |
| *P. vlangalii* | *P. vlangalii pylzowi* | MK461401 | ZMMU R-8899_1 | China, Xinjiang, Chemo (= Cherchen, Tzemo), Altyntag mts., Chinbulak, 60 km S Tura, Cherchen bank | 87.7 | 37.9 | Solovyeva *et al.* 2023 |
| *P. vlangalii* | *P. vlangalii pylzowi* | HM362988 | CIB XM3540 | China, Sichuan, Ruoergai | 102.95 | 33.87 | Gu *et al.* 2011 |
| *P. vlangalii* | *P. vlangalii pylzowi* | KF691719 | KIZ 020062 | China, Qinghai, Gonghe [vicinity of the type locality of vlangalii] | 99.546 | 35.627 | Solovyeva *et al.* 2014 |
| *P. vlangalii* | *P. vlangalii pylzowi* | MK492321 | ZMMU R-8897_1 | China, Xinjiang, Chemo (= Cherchen, Tzemo), Altyntag mts., Minbulak, left Cherchen bank, Achik-kul Depression | 87.7 | 37.9 | Solovyeva *et al.* 2023 |
| *P. vlangalii* | *P. vlangalii pylzowi* | MK492320 | ZMMU R-8898_1 | China, Xinjiang, Chemo (= Cherchen, Tzemo), Middle Kunlun, Achik-kul Depression, Minbulak | 87.7 | 37.9 | Solovyeva *et al.* 2023 |
| *P. vlangalii* | *P. vlangalii pylzowi* | MK492322 | ZMMU R-14325 | China, Qinghai, Tasan Nur | 98.761 | 35.264 | Solovyeva *et al.* 2023 |
| *P. vlangalii pylzowi* | *P. vlangalii pylzowi* | MF039059 | - | China, Maqu Xian, Gannan Tibetan Autonomous Prefecture, Gansu Province | 102.90 | 34.96 | Jin *et al.* 2018 |
| *P. vlangalii pylzowi* | *P. vlangalii pylzowi* | MF039060 | - | China: Maduo County, Tibetan Autonomous Prefecture of Golog, Qinghai Province | 98.06 | 34.44 | Jin *et al.* 2018 |
| *P. hongyuanensis* | *P. vlangalii* | AY053821 | CIB0541 | Hongyuan Prefecture, Sich_x0002_uan Province, P.R. China | - | - | Pang *et al.* 2003 |
| *P. hongyuanensis* | *P. vlangalii* | AY053820 | CIB0714 | Hongyuan Prefecture, Sich_x0002_uan Province, P.R. China | - | - | Pang *et al.* 2003 |
| *P. vlangalii* | *P. vlangalii* | AY053873 | CIB0793 | DulanPrefecture, Qinghai Province, China | - | - | Pang *et al.* 2003 |
| *P. vlangalii* | *P. vlangalii* | AY053876 | CIB0740 | Aksay Prefec_x0002_ture, Gansu Province, China | - | - | Pang *et al.* 2003 |
| *P. vlangalii* | *P. vlangalii* | AY053875 | CIB0797 | Gol mud Prefecture, Qinghai Province, KIZ-Rdq1-3;Xiangride Farm, Qinghai Province, China | - | - | Pang *et al.* 2003 |
| *P. vlangalii* | *P. vlangalii* | AY053874 | CIB0796 | Gol mud Prefecture, Qinghai Province, KIZ-Rdq1-4;Xiangride Farm, Qinghai Province, China | - | - | Pang *et al.* 2003 |
| *P. vlangalii* | *P. vlangalii* | AY053878 | CIB0972 | Suhai Lake,Qinghai Province, China | - | - | Pang *et al.* 2003 |
| *P. vlangalii* | *P. vlangalii* | AY053877 | CIB0970 | Suhai Lake,Qinghai Province, China | - | - | Pang *et al.* 2003 |
| *P. theobaldi orientalis* | *P. lhasaensis* | KJ551842 | - | China, Tibet, Xizang, environs of Rutog [vicinity of the type locality of lhasaensis] | 80.31 | 33.68 | Liao & Jin 2016 |
| *P. theobaldi* | *P. theobaldi* | AY053856 | CIB7495 | China, Mailing Prefecture, Tibet Autonomous Region | - | - | Pang *et al.* 2003 |
| *P. theobaldi* | *P. theobaldi* | AY053854 | CIB0070 | Lhasa, Tibet Autonomous Re_x0002_gion, P.R. China | - | - | Pang *et al.* 2003 |
| *P. theobaldi* | *P. theobaldi* | AY053855 | CIB0071 | Lhasa, Tibet Autonomous Re_x0002_gion, P.R. China | - | - | Pang *et al.* 2003 |
| *P. zetangensis* | *P. theobaldi* | AY053883 | KIZ-Rdq20 | Zetang Prefecture, Tibet Au_x0002_tonomous Region, P.R. China, | - | - | Pang *et al.* 2003 |
| *P. zetangensis* | *P. theobaldi* | AY053882 | CIB7594 | Zetang Prefecture, Tibet Au_x0002_tonomous Region, P.R. China, | - | - | Pang *et al.* 2003 |
| *P. theobaldi* | *P. theobaldi* | KF691721 | ZMMU R-10816 | India, Djammu-and-Kashmir, Ladakh, Rupshu river, Teokar valley [type locality of theobaldi] | 77.99 | 33.01 | Solovyeva *et al.* 2014 |
| *P. theobaldi* | *P. theobaldi* | MK461380 | ZMMU R-12139-2 | India, Jammu-Kashmir, Ladakh, Rupshu river, Teokar valley | 77.99 | 33.01 | Solovyeva *et al.* 2023 |
| *P. theobaldi* | *P. theobaldi* | MK461379 | ZMMU R-12139-1 | India, Jammu-Kashmir, Ladakh, Rupshu river, Teokar valley | 77.99 | 33.01 | Solovyeva *et al.* 2023 |
| *P. theobaldi* | *P. theobaldi* | MK461376 | ZMMU R-12138-1 | India, Jammu-Kashmir, Ladakh | 77.07 | 33.02 | Solovyeva *et al.* 2023 |
| *P. theobaldi* | *P. theobaldi* | KF691723 | ZMMU R-12138-2 | India, Jammu-Kashmir, Ladakh | 77.07 | 33.02 | Solovyeva *et al.* 2014 |
| *P. theobaldi theobaldi* | *Phrynocephalus sp. 1* | MF039063 | - | China, Tibet, Ngari Prefecture | 80.25 | 33.12 | Jin & Brown 2018 |
| *Phrynocephalus sp. 2* | *Phrynocephalus sp. 2* | KF691720 | ZMMU R-12301 | China, Tibet, Xizang, 20 km from Pelguzo Lake | 85.879 | 28.665 | Solovyeva *et al.* 2014 |
| *P. theobaldi orientalis* | *Phrynocephalus sp. 2* | MF039062 | - | China, Tibet, Brahmaputra River valley | 83.49 | 30.03 | Jin *et al.* 2018 |
| *P. parvus* | *P. parvus* | KJ630904 | - | China, Qinhai, Tuotuo River (Ulan Moron)[vicinity of the type locality of parvus] | 92.91 | 34.09 | Zhu *et al.* 2016 |
| *P. erythrurus erythrurus* | *P. erythrurus* | MF039065 | - | China, Amdo, Southern Qiangtang Plateau of QTP | 91.58 | 32.15 | Jin *et al.* 2018 |
| *P. guinanensis* | *P. putjatai* | KJ885621 | - | China, Guinan county, Qinghai Province [type locality of guinanensis] | - | - | Fu *et al.* 2016 |
| *P. putjatai* | *P. putjatai* | KJ830752 | - | China, Nanhai Temple, Guide county, Qinghai Province [type locality of putjatai] | - | = | Tong & Jin 2016 |
| *P. putjatai* | *P. putjatai* | MK461405 | ZMMU R-12308-2 | China, Qinghai, Qinhai-Tibet plateu, N coast of the Kukunor Lake, N coast of the Kukunor Lake | 100.75 | 37.28 | Solovyeva *et al.* 2023 |
| *P. putjatai* | *P. putjatai* | KF691722 | KIZ 020238 | China, Qinghai, env. of. Qinghai Lake | 100.79 | 36.72 | Solovyeva *et al.* 2014 |
| *P. putjatai* | *P. putjatai* | MK461411 | ZMMU R-12308-1 | China, Qinghai, Qinghay-Tibet table, NE border of Kukunor (Qinghay) Lake | 100.75 | 37.28 | Solovyeva *et al.* 2023 |
| *P. axillaris* | *P. axillaris* | AY053779 | CIB0989 | Aksay Prefecture, Gansu Province,China | - | - | Pang *et al.* 2003 |
| *P. axillaris* | *P. axillaris* | MK284224 | WGXG08226 | China, Turpan Desert Botanical Garden | - | - | Chen *et al.* 2019b |
| *P. mystaceus* | *P. mystaceus* | AY053822 | MVZ-TP22375 | Ili Prefecture, Xinjiang UygurAutonomous Region, P.R. China | - | - | Pang *et al.* 2003 |
| *P. mystaceus* | *P. mystaceus* | KF691713 | ZMMU R-12261 | Uzbekistan, Navoi, Yamankum Desert, 3 - 4 W from Aktakyr | 64.02 | 41.74 | Solovyeva *et al.* 2018 |

**References**

Chen, D., Li, J., & Guo, X. 2019a. Next-generation sequencing yields a nearly complete mitochondrial genome of the Forsyth’s toad-headed agama, *Phrynocephalus forsythii* (Reptilia, Squamata, Agamidae). *Mitochondrial DNA Part B*, 4(1), 817-819. DOI: 10.1080/23802359.2019.1574681.

Chen, D., Li, J., & Guo, X. 2019b. Next-generation sequencing yields a nearly complete mitochondrial genome of the Yarkand toad-headed agama (*Phrynocephalus axillaris*) from the Turpan Depression. *Mitochondrial DNA Part B*, 4(1), 1198-1199. DOI: 10.1080/23802359.2019.1591228.

Chen, D., Zhou, T., & Guo, X. 2016. The complete mitochondrial genome of *Phrynocephalus forsythii* (Reptilia, Squamata, Agamidae), a toad-headed agama endemic to the Taklamakan Desert. *Mitochondrial DNA Part A*, 27(6), 4046-4048. DOI: 10.3109/19401736.2014.1003837.

Fu, C., Chen, W., & Jin, Y. 2016. The complete mitochondrial genome of *Phrynocephalus guinanensis* (Reptilia, Squamata, Agamidae). *Mitochondrial DNA Part A*, 27(2), 1103-1104. DOI: 10.3109/19401736.2014.933320.

Gu, H. F., Xia, Y., Peng, R., Mo, B. H., Li, L., & Zeng, X. M. 2011. Authentication of Chinese crude drug gecko by DNA barcoding. *Natural product communications*, 6(1), 1934578X1100600117. DOI: 10.1177/1934578X1100600117.

Guo, X.; Liu, J.; Chen, M.; Xu, R. 2022. *Phrynocephalus nasatus* Mitochondrion, Complete Genome. Available online: https://www.ncbi.nlm.nih.gov/nuccore/MW456559.1 (accessed on 1 July 2022).

Jin, Y., & Brown, R. P. 2018. Partition number, rate priors and unreliable divergence times in Bayesian phylogenetic dating. *Cladistics*, 34(5), 568-573. DOI: 10.1111/cla.12223.

Liao, P., & Jin, Y. 2016. The complete mitochondrial genome of the toad-headed lizard subspecies, *Phrynocephalus theobaldi orientalis* (Reptilia, Squamata, Agamidae). *Mitochondrial DNA Part A*, 27(1), 559-560. DOI: 10.3109/19401736.2014.905857.

Pang J, Wang Y, Zhong Y, Hoelzel AR, Papenfuss TJ, Zeng X, Ananjeva NB, & Zhang, Y. P. 2003. A phylogeny of Chinese species in the genus *Phrynocephalus* (Agamidae) inferred from mitochondrial DNA sequences. Molecular phylogenetics and evolution, 27(3), 398-409. DOI: 10.1016/S1055-7903(03)00019-8.

Shao, M., Ma, L., & Wang, Z. 2016. The complete mitochondrial genome of the toad-headed lizard, *Phrynocephalus forsythii* (Reptilia, Squamata, Agamidae). *Mitochondrial DNA Part A*, 27(5), 3147-3148.

Solovyeva, E. N., Poyarkov, N. A., Dunaev, E. A., Duysebayeva, T. N., & Bannikova, A. A. 2011. Molecular differentiation and taxonomy of the sunwatcher toad-headed agama species complex *Phrynocephalus* superspecies *helioscopus* (Pallas 1771)(Reptilia: Agamidae). *Russian Journal of Genetics*, 47, 842-856.

Solovyeva, E. N., Poyarkov, N. A., Dunayev, E. A., Nazarov, R. A., Lebedev, V. S., & Bannikova, A. A. 2014. Phylogenetic relationships and subgeneric taxonomy of toad-headed agamas *Phrynocephalus* (Reptilia, Squamata, Agamidae) as determined by mitochondrial DNA sequencing. *In Doklady Biological Sciences* (Vol. 455, No. 1, p. 119). Springer Nature BV. DOI: 10.1134/S0012496614020148.

Solovyeva, E. N., Dunayev, E. A., Nazarov, R. A., Bondarenko, D. A., & Poyarkov, N. A. 2023. COI-barcoding and species delimitation assessment of toad-headed Agamas of the genus *Phrynocephalus* (Agamidae, Squamata) reveal unrecognized diversity in Central Eurasia. *Diversity*, 15(2), 149.

Solovyeva, E. N., Dunayev, E. N., Nazarov, R. A., Radjabizadeh, M., & Poyarkov, N. A. 2018. Molecular and morphological differentiation of Secret Toad-headed agama, *Phrynocephalus mystaceus*, with the description of a new subspecies from Iran (Reptilia, Agamidae). *ZooKeys*, (748), 97. DOI: 10.3897/zookeys.748.20507.

Tong, H., & Jin, Y. 2016. The complete mitochondrial genome of an agama, Phrynocephalus putjatia (Reptilia, Squamata, Agamidae). *Mitochondrial DNA Part A*, 27(2), 1028-1029. DOI: 10.3109/19401736.2014.926538.

Zhu, L., Liao, P., Tong, H., & Jin, Y. 2016. The complete mitochondrial genome of the subspecies, *Phrynocephalus erythrurus parva* (Reptilia, Squamata, Agamidae), a toad-headed lizard dwell at highest elevations of any reptile in the world. *Mitochondrial DNA Part A*, 27(1), 703-704. DOI: 10.3109/19401736.2014.913151.
